# Changes in the Soil Microbiome in Eggplant Monoculture Revealed by High-Throughput Illumina MiSeq Sequencing as Influenced by Raw Garlic Stalk Amendment

**DOI:** 10.3390/ijms20092125

**Published:** 2019-04-29

**Authors:** Muhammad Imran Ghani, Ahmad Ali, Muhammad Jawaad Atif, Muhammad Ali, Bakht Amin, Muhammad Anees, Haris Khurshid, Zhihui Cheng

**Affiliations:** 1 College of Horticulture, Northwest A&F University, Yangling 712100, China; imran_pak@nwsuaf.edu.cn (M.I.G.); Ahmadhort87@nwafu.edu.cn (A.A.); jawaadatif@nwafu.edu.cn (M.J.A.); muhammadali@nwsuaf.edu.cn (M.A.); Bakhtamin96@nwafu.edu.cn (B.A.); 2 Vegetable Crops Program, National Agricultural Research Centre, Islamabad 44000, Pakistan; 3 Department of Microbiology, Kohat University of Science & Technology, Kohat 26000, Pakistan; dr.anees@kust.edu.pk; 4 Oilseeds Research Program, National Agricultural Research Centre, Islamabad 44000, Pakistan; hariskhurshid8@gmail.com

**Keywords:** eggplant, monoculture, microbial community composition, garlic stalk amendments

## Abstract

The incorporation of plant residues into soil can be considered a keystone sustainability factor in improving soil structure function. However, the effects of plant residue addition on the soil microbial communities involved in biochemical cycles and abiotic stress phenomena are poorly understood. In this study, experiments were conducted to evaluate the role of raw garlic stalk (RGS) amendment in avoiding monoculture-related production constraints by studying the changes in soil chemical properties and microbial community structures. RGS was applied in four different doses, namely the control (RGS0), 1% (RGS1), 3% (RGS2), and 5% (RGS3) per 100 g of soil. The RGS amendment significantly increased soil electrical conductivity (EC), N, P, K, and enzyme activity. The soil pH significantly decreased with RGS application. High-throughput Illumina MiSeq sequencing revealed significant alterations in bacterial community structures in response to RGS application. Among the 23 major taxa detected, Anaerolineaceae, *Acidobacteria*, and *Cyanobacteria* exhibited an increased abundance level. RGS2 increased some bacteria reported to be beneficial including *Acidobacteria*, *Bacillus*, and *Planctomyces* (by 42%, 64%, and 1% respectively). Furthermore, internal transcribed spacer (ITS) fungal regions revealed significant diversity among the different treatments, with taxa such as *Chaetomium* (56.2%), *Acremonium* (4.3%), *Fusarium* (4%), *Aspergillus* (3.4%), Sordariomycetes (3%), and Plectosphaerellaceae (2%) showing much abundance. Interestingly, *Coprinellus* (14%) was observed only in RGS-amended soil. RGS treatments effectively altered soil fungal community structures and reduced certain known pathogenic fungal genera, i.e., *Fusarium* and *Acremonium*. The results of the present study suggest that RGS amendment potentially affects the microbial community structures that probably affect the physiological and morphological attributes of eggplant under a plastic greenhouse vegetable cultivation system (PGVC) in monoculture.

## 1. Introduction

The application of crop residues is of great importance due to its positive effect on both crop and soil health [1]. In addition to preserving the agro-ecosystem, the incorporation of crop residues generally results in increased soil organic matter and improved defense against destructive pathogens [2] and essential nutrient availability [3,4]. Moreover, the application of residues with different doses has shown different effects on soil fungal and bacterial diversity [5]. It was reported that the incorporation of *Brassica* residues in consecutive monocropping of eggplant not only increased plant growth but also acted as a bio-control agent against numerous plant pathogens [6]. As a sustainable and environmentally friendly approach, the addition of different plant residues containing essential oils such as terpenes, alcohols, and phenolic compounds with biocidal activities has been recommended to decrease pathogen incidence in monocultured soils [7].

The raw garlic stalk (RGS) is a byproduct of garlic and, as it is produced abundantly after harvesting crops, it is usually left on the soil or burnt, causing air pollution. China is currently a leading producer of garlic in the world and is responsible for 25% to 30% of total garlic production [8,9]. We recently reported the beneficial impact of the incorporation of RGS as a soil amendment on plant growth and antioxidant systems. Moreover, RGS is a good option due to its easy availability, low cost, and eco-friendly nature [10]. RGS incorporation may lead to the production of thiosulfinates, disulfides, and other related soil phytochemicals with known antimicrobial activities against some plant pathogens, bearing equivalent effects to fungicides such as methyl bromide [11]. We also recently reported the impact of RGS on the development and yield of eggplant in monoculture under plastic greenhouse conditions; however, there are no reports regarding the in situ impact of RGS incorporation on the soil chemical properties and microbial community structures that may be instrumental in defining the outcome of the residue incorporation [10].

Microorganisms are the most important living entities; they not only affect the health of soil and nutrient availability, but also play important roles in maintaining crop growth and yield [12]. These microorganisms influence the rhizosphere ecosystem and also act as natural antagonists against plant pathogens. Researchers have reported the role of certain bacterial species such as *Bacillus*, *Paenibacillus*, and *Pseudomonas* as well as fungal species such as *Trichoderma*, *Gliocladium*, and *Phomopsis* acting as bio-control agents against *Fusarium* wilt [13,14]. Long-term continuous cropping drastically influences soil nutrient composition, resulting in soil acidification and salinization, which subsequently affects overall plant production [15]. The reduction in plant yield has been attributed to auto toxicity, a form of allelopathy, which is triggered by replanting failure in the form of releasing phytotoxic allelochemicals, imparting negative effects on the plant growth of the same plant species in the coming seasons [16,17]. Research studies have also confirmed the allelopathic effects in continuous cropping systems in different agronomic crops, such as the wheat–rice–maize intensive cropping system [18,19]. However, such reports are scarce in horticulture crops. Continuous cropping and autotoxicity affect the soil microbiome [17]. Therefore, the plant–soil microbiome interaction as a result of continuous cropping, and subsequent autotoxicity can be transformed with different crops such as peach, cucumber [20], lily turf [21], and female ginseng [17].

The garlic stalk residue amendment is expected to improve the plant growth and fruit productivity as reported in our previous study [10]. However, little information exists on the dynamism of soil quality changes, or their influence on the core soil microbiome structure revealed by metagenomic analysis in the rhizosphere soil of a continuous eggplant cropping system. Consequently, the aim of this study was to check the dose-dependent effect of RGS for soil chemical, biological, and microbial abundance. We hypothesized that incorporation of RGS would result in significant changes in soil biology structure and thereby effect the temporal microflora variability. For this purpose, the dose-dependent effect was investigated in pot greenhouse experiments over two years of RGS application.

## 2. Results

### 2.1. Effect of RGS on Soil Chemical Properties

The incorporation of RGS had a considerable impact on soil pH and the electrical conductivity (EC) of eggplant monocultured soil over both years (Table 1). Soil pH exhibited a decline with RGS incorporation as compared to the control in both years, 2016 and 2017. The highest soil pH decline was recorded in RGS3 in both years, as compared to the control. Contrarily, soil EC values showed an increasing trend with the addition of RGS in both years. The highest value was found in the treatment application of RGS3 in 2016 (234.00 µs·cm^−1^) and 2017 (238.67 µs·cm^−1^).

The contents of soil organic carbon and organic matter were also increased by RGS application. A higher amount of applied raw garlic stalk attained the maximum organic carbon retention with a significant proportion of 51.60% in 2016 and 50.08% in 2017. This trend was uniform for the addition of soil organic matter to the soil (Table 1). Differences between the pH, EC, organic carbon, and organic matter of treatments were significant, while between years and treatment interactions, non-significant differences were exhibited (Table 1).

Available soil N, P, and K were significantly higher in RGS-amended soil as compared to the control (RGS0) during both years. RGS3 showed highest available N, P, and K with percent increases of 69%, 14%, and 297% in 2016, displaying similar trends in 2017 as well. It was observed that the year and treatment had non-significant effects on these parameters (Table 2). RGS amendment enhanced the soil invertase activity in all treatments as compared to RGS0. The highest invertase activity was observed in RGS3 during both years, with 45% and 47% increases during 2016 and 2017, respectively, as compared to the controls (Table 3). Urease and alkaline phosphatase activities exhibited similar trends. RGS amendment also significantly increased dehydrogenase activity, reaching the highest values in RGS2 (3:100 g), reflecting 57% and 59% increases in 2016 and 2017, respectively, compared to the control. Soil enzymes showed non-significant differences among the different treatments and between the two years, apart from urease activity.

### 2.2. Soil Microbe Identifications

The molecular characterization for microbial profiling indicated a total of 31,950 reads in the barcode of the 515F and 907R regions of the bacterial 16S rRNA gene, and 50,540 reads in the internal transcribed spacer (ITS) fungal region were identified across all samplings. In the bacterial population, we identified 3756 operational taxonomic units (OTUs) that were further classified as 33 phyla, 80 classes, 190 orders, 379 families, 750 genera, and 1418 species. Likewise, 50,540 fungal reads yielded a total of 470 OTUs, which were classified as 9 phyla, 21 classes, 40 orders, 80 families, 139 genera, and 212 species.

### 2.3. Soil Microbial Diversity

The incorporation of RGS reduced the bacterial richness estimator (Ace and Chao), but increased the diversity estimator (Simpson and Shannon) in RGS2 and RGS3, respectively, as shown in Table S1. However, fungal richness and the diversity estimator increased in RGS2 except for the Simpson diversity, as compared to RGS0. For bacteria, the rarefaction curves of each treatment showed a flat trend, indicating that the soil sampling effort was sufficient to capture the true richness of the bacterial and fungal community.

### 2.4. Venn Diagram Analysis

**Figure 1:**
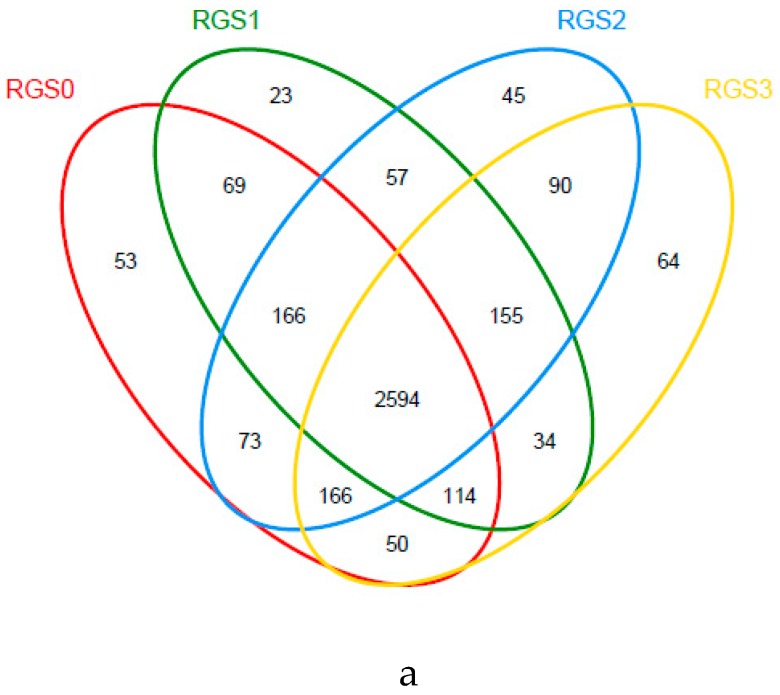

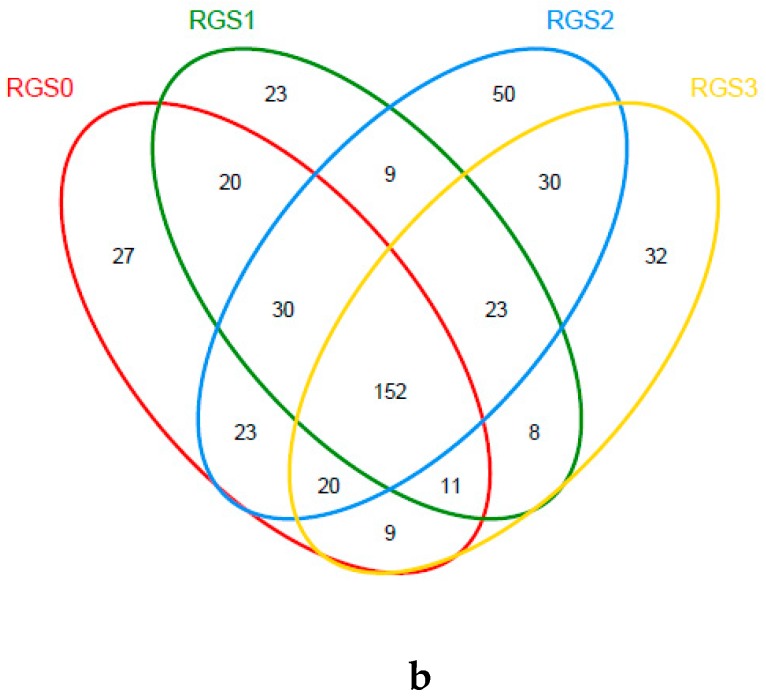
Venn diagram based on operational taxonomic units (OTUs) of bacterial (**a**) and fungal (**b**) community with different raw garlic stalk (RGS) treatments.

A Venn diagram was used to show the treatment-wise distribution of common and unique OTU subsets of bacteria and fungi (Figure 1a,b). A total of 2594 bacterial OTUs were found to be common among all the treatments (Figure 1a). Most of the subsets (3005, 45.44%) were identical between RGS2 and RGS3, while RGS3 had (64, 1.95%) the highest number of unique OTUs, followed by RGS0 (53, 1.61%), RGS2 (45, 1.34%), and RGS1 (23, 0.70%). Similarly, 152 fungal OTUs were common in the soil of different treatments (Figure 1b). RGS2 had the maximum number of unique OTUs (50, 14.83%), followed by RGS3 (32, 11%), RGS0 (27, 9.24%), and RGS1 (23, 8.33%). The maximum number of OTUs was shared between RGS2 and RGS3 treatments. Hence, on the basis of similar OTUs, RGS0 was nearly identical to RGS1, while RGS2 and RGS3 with higher concentrations of raw garlic stalk had almost same fungal population.

### 2.5. Changes in Bacterial and Fungal Community

**Figure 2:**
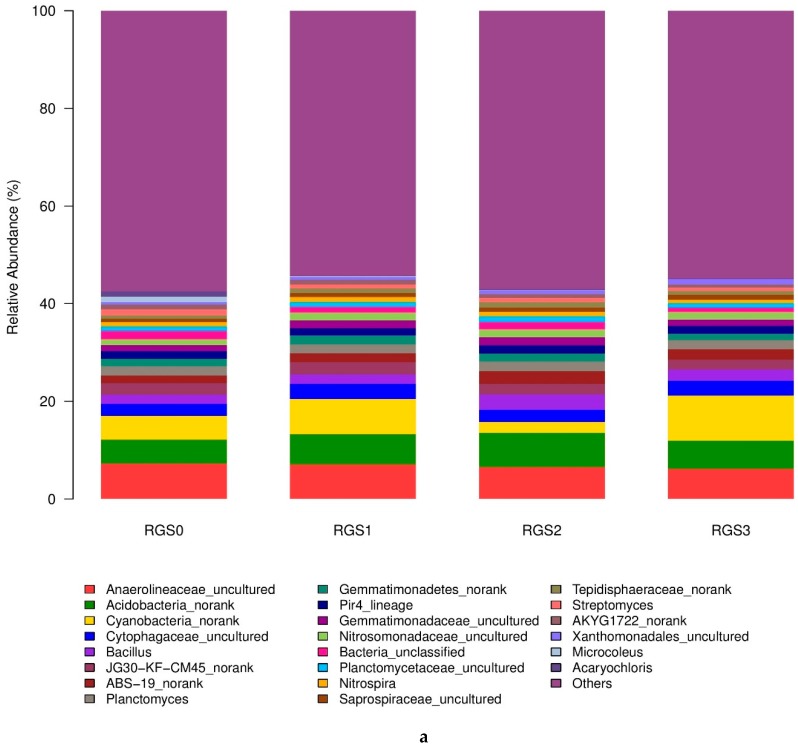

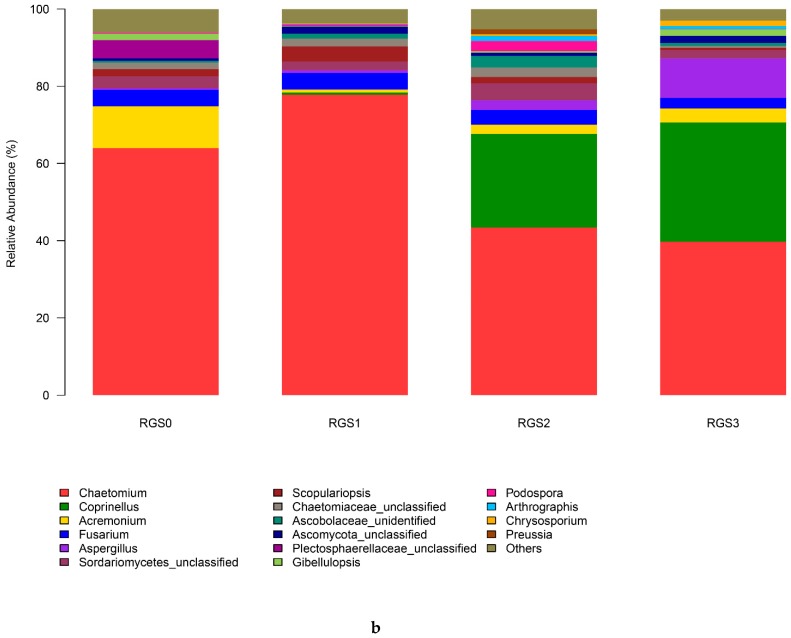
Relative abundance of different bacterial (**a**) and fungal (**b**) taxa in all the treatments.

The composition and abundance of various taxonomic groups varied among the treatments. The relative abundance of different taxa in different treatments is shown (Figure 2a,b). The effect of RGS incorporation could influence the phylogenetic composition and community structure with different levels of application. Notably, the application of RGS changed the bacterial community structures. Anaerolineaceae, Acidobacteria, and Cyanobacteria were the most common and abundant groups, having abundance levels of 6.7%, 6%, and 5%, respectively. RGS application increased the relative abundance of Acidobacteria, Cytophagaceae, *Bacillus*, Nitrosomonadaceae, and Xanthomonadales. The application of RGS1 and RGS2 relatively increased the abundance of *Azoarcus* (59% and 5%) and Gemmatimonadetes (19% and 7%), respectively. Meanwhile, a higher amount of RGS3 decreased the relative abundance of these bacterial taxa. RGS1 and RGS3 increased the relative abundance of Cyanobacteria (46% and 88%) and *Steroidobacter* (11% and 1%), respectively, as compared to non-amended soil. The application of RGS had an inhibitory effect on the proliferation of Anaerolineaceae and *Streptomyces* as compared to unamended soil. The application of RGS also had an inhibitory effect on the proliferation of Anaerolineaceae and *Streptomyces* at the final time point of the analysis.

At the fungal level, seven major fungal taxa, i.e., *Chaetomium* (56.2%), *Coprinellus* (14%), *Acremonium* (4.3%), *Fusarium* (4%), *Aspergillus* (3.4%), Sordariomycetes (3%), and Plectosphaerellaceae (2%), were found in highly abundant proportions, while the 10 remaining taxa showed the lowest abundances—less than 1% in most of the treatment samples (Figure 2a,b). The tested treatments revealed a variable response across fungal taxa compositions. RGS0 had a higher abundance of *Chaetomium* (64%), followed by *Acremonium* (10.8%), Plectosphaerellaceae (4.78%), *Fusarium* (4.3%), Sordariomycetes (3.1%), *Scopulariopsis* (1.7%), Chaetomiaceae (1.7%), and *Gibellulopsis* (1.6%); the remaining fungal taxa had abundance levels of less than 1%. In RGS1, a significant increase was observed in the abundance level of *Chrysosporium* (877%), Ascomycota (211%), Ascobolaceae (131%), *Scopulariopsis* (122%), *Aspergillus* (94%), and *Chaetomium* (20%) as compared to RGS0. Likewise, a considerable decline in the abundance level of *Acremonium* (92%), Plectosphaerellaceae (99%), *Gibellulopsis* (90%), and Sordariomycetes (28%) was recorded. In addition, *Coprinellus* was detected in RGS1 but was absent in RGS0. In RGS2, *Podospora*, *Arthrographis*, *Chrysosporium*, *Preussia*, and *Aspergillus* increased as compared to RGS0. Furthermore, a sharp reduction was observed for *Chaetomium* (32%), *Acremonium* (77%), *Fusarium* (11%), Plectosphaerellaceae (100%), and *Gibellulopsis* (77%). RGS3 had several-fold higher abundance levels of *Arthrographis*, *Chrysosporium*, and *Aspergillus* as compared to RGS0. The genus *Coprinellus* constituted 30% of the RGS3 fungal community, with the highest level among all the treatments. Similarly, Ascobolaceae and Ascomycota also increased by 49% and 209% in the soil amended with RGS3. Conversely, a significant decrease in the abundance level of some important pathogenic fungal taxa, i.e., Chaetomiaceae (73%), *Scopulariopsis* (67%), *Acremonium* (66%), *Fusarium* (35%), and Sordariomycetes (28%), was observed.

### 2.6. Linear Discriminant Analysis Effect Size (LEfSE) Analysis of Microbial Community

**Figure 3:**
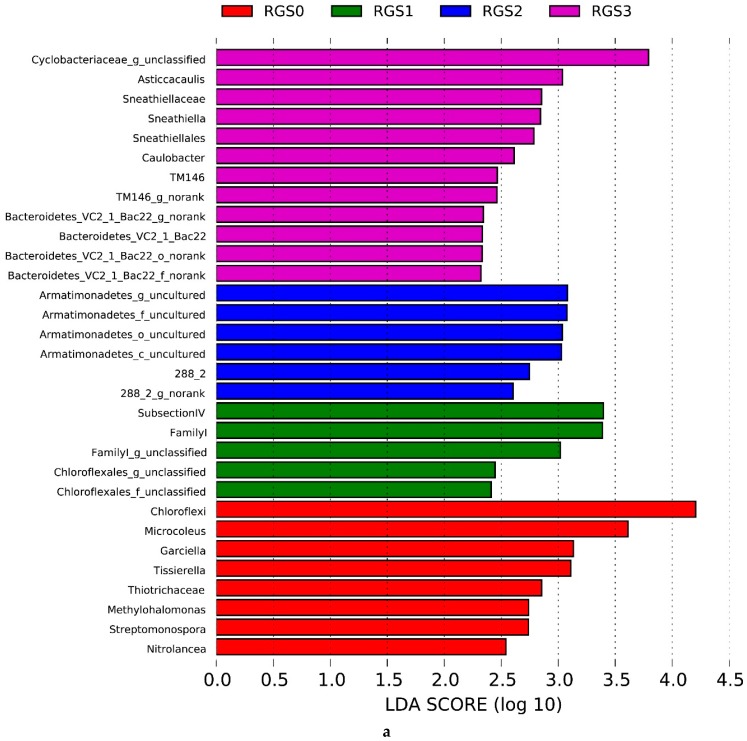

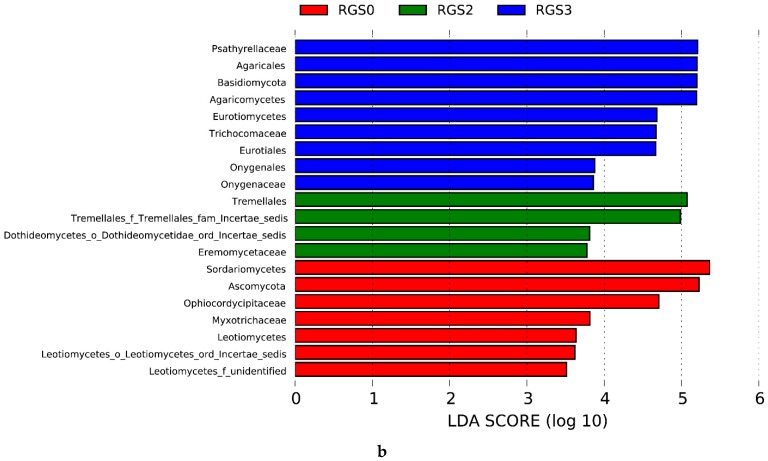
Linear discriminant analysis (LDA) score showing significantly different bacterial (**a**) and fungal (**b**) groups in treatments.

**Figure 4:**
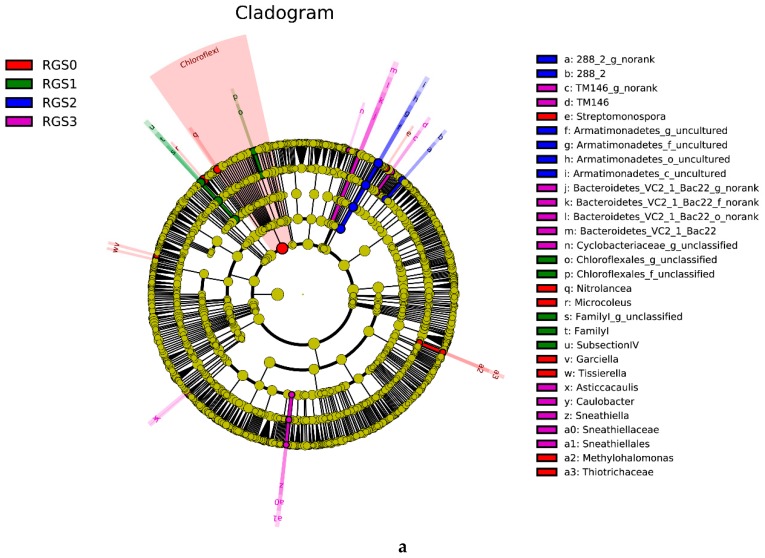

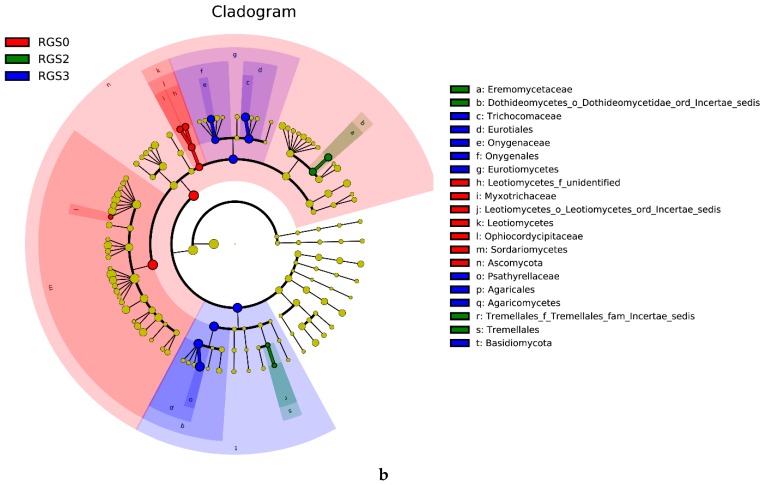
Linear discriminant analysis effect size (LEfSE) cladogram showing differences of bacterial (**a**) and fungal (**b**) community structure between different RGS treatments. The black circles from center to outer represent phylum, class, order, family, genus, and species. Red, green, blue and purple circles show taxa that were abundant in the RGS0, RGS1, RGS2, and RGS3, respectively.

Linear discriminant analysis effect size (LEfSE) is an efficient statistical analysis for high-efficiency biomarker discovery that enables the detailed identification of abundant features by characterizing potential discriminating taxa between different biological groups. A linear discriminant analysis (LDA) score higher than 2 was used to discriminate only those bacterial and fungal groups that were significantly different between the four treatments, i.e., RGS0 to RGS3 (Figure 3a,b). A cladogram depicted all the treatment-wise taxa with LDA values higher than 2.5 (Figure 4a,b). Major bacterial groups identified in RGS0 were *Chloroflexi*, *Microcoleus*, *Garciella*, *Tissierella*, Thiotrichaceae, *Methylohalomonas*, *Streptomonospora*, and *Nitrolancea*. RGS1 had a higher abundance of *Family I* and Chloroflexales. RGS2 had a higher level of Armatimonadetes while RGS3 had a higher abundance of Cyclobacteriaceae, *Asticcacaulis*, Sneathiellaceae, *Sneathiella*, Sneathiellales, *Caulobacter*, *TM146*, and Bacteroidetes.

For fungal communities, an LDA score higher than 2 was used as the criterion indicating the presence of the most abundant taxa in RGS0, RGS2, and RGS3, whereas RGS1 was devoid of uniquely abundant fungal taxa and was hence excluded as a distinct group. RGS0 included Eremomycetaceae, Sordariomycetes, Ascomycota, Ophiocordycipitaceae, Myxotrichaceae, and Leotiomycetes as highly abundant taxa. RGS2 had a high abundance of Tremellales, Eremomycetaceae, and Dothideomycetes, while RGS3 had higher contents of Psathyrellaceae, Agaricales, *Basidiomycota*, Agaricomycetes, Eurotiomycetes, Trichocomaceae, Eurotiales, Onygenales, and Onygenaceae.

### 2.7. Top Abundant Microbial Distribution Revealed by Heat Map Analysis

**Figure 5:**
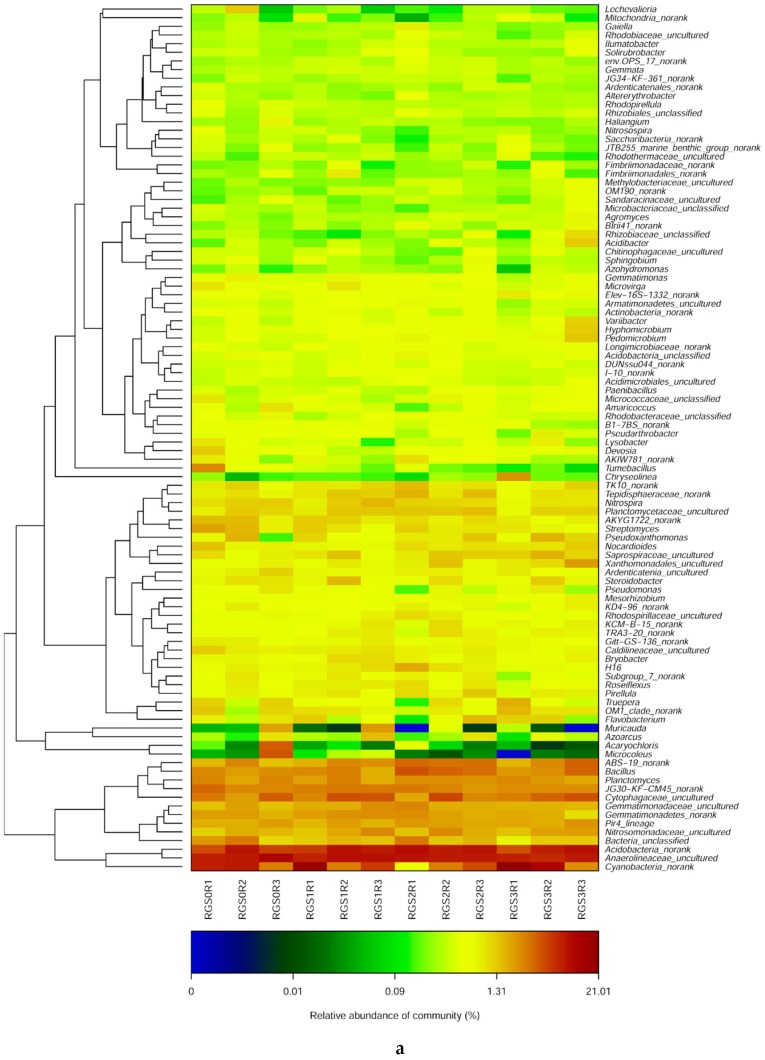

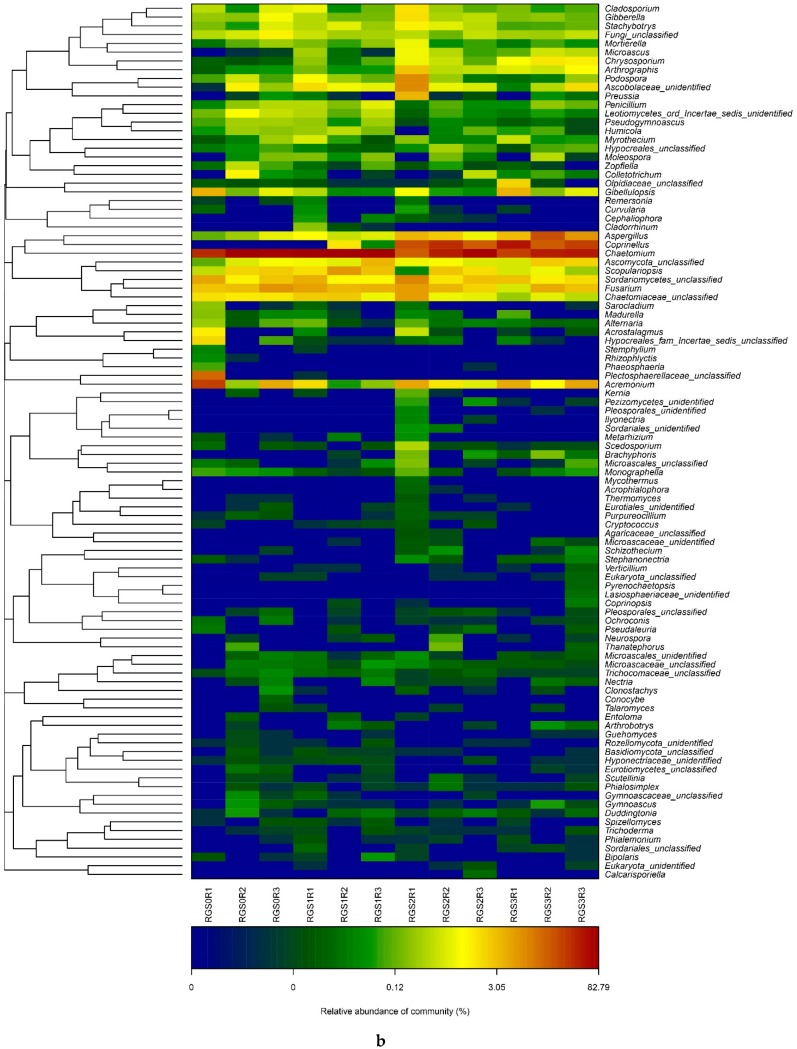
Heatmap analysis showing bacterial (**a**) and fungal (**b**) community distribution in all the treatments.

Furthermore, heat map analysis was performed to visualize the distribution of microbial communities in different RGS treatments (Figure 5a,b). The entire set of bacterial communities observed through sequence analyses were divided into four clusters, where each treatment was considered as a group. RGS0 and RGS1 were clustered in the same group, while RGS2 and RGS3 were clustered together based on similar bacterial community structures. Further, the bacterial populations were classified according to phylogenetic and relative abundance and illuminated by colors. RGS0 had a higher abundance of AKYG1722, *Streptomyces*, and Anaerolineaceae. *Acaryochloris* was lower in RGS1 and completely absent in RGS3. Likewise, RGS2 and RGS3 were completely devoid of *Microcoleus*.

### 2.8. Principal Component Analysis (PCA)

**Figure 6:**
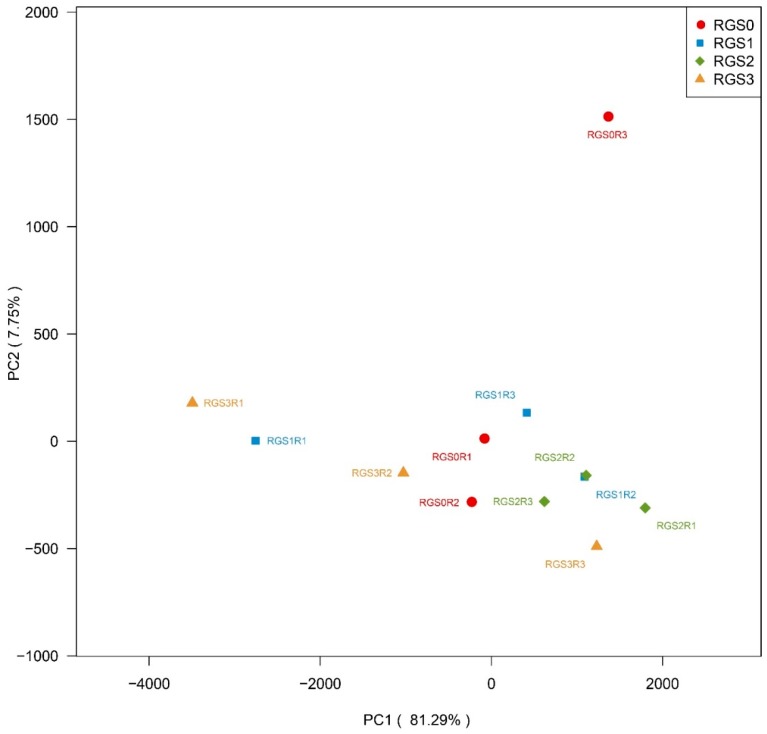
Principal component analysis (PCA) scatter plot showing first two principal components based variation among replication of different RGS treatments.

PCA analysis was performed for all 3756 bacterial OTUs detected in the four treatments, including RGS0 distributing total variation in 12 components. The first six principal components (PCs) had eligen values higher than unity (>1), but only PC1 and PC2 were selected to explain the variation present in the treatments. These PCs collectively contributed 89.04% variation and were plotted on a biplot (Figure 6). On the basis of similarity between bacterial communities, RGS2 replications were identical and were clustered together. The third replication of RGS0 was unique and settled in the first quadrant, unlike the majority of the treatment’s replications, which remained in the third and fourth quadrants. The distribution of replications showed that RGS2 and RGS3 were similar vis-à-vis OTU distribution.

### 2.9. Hierarchical Cluster Analysis of Fungal Community in RGS Treatments

**Figure 7:**
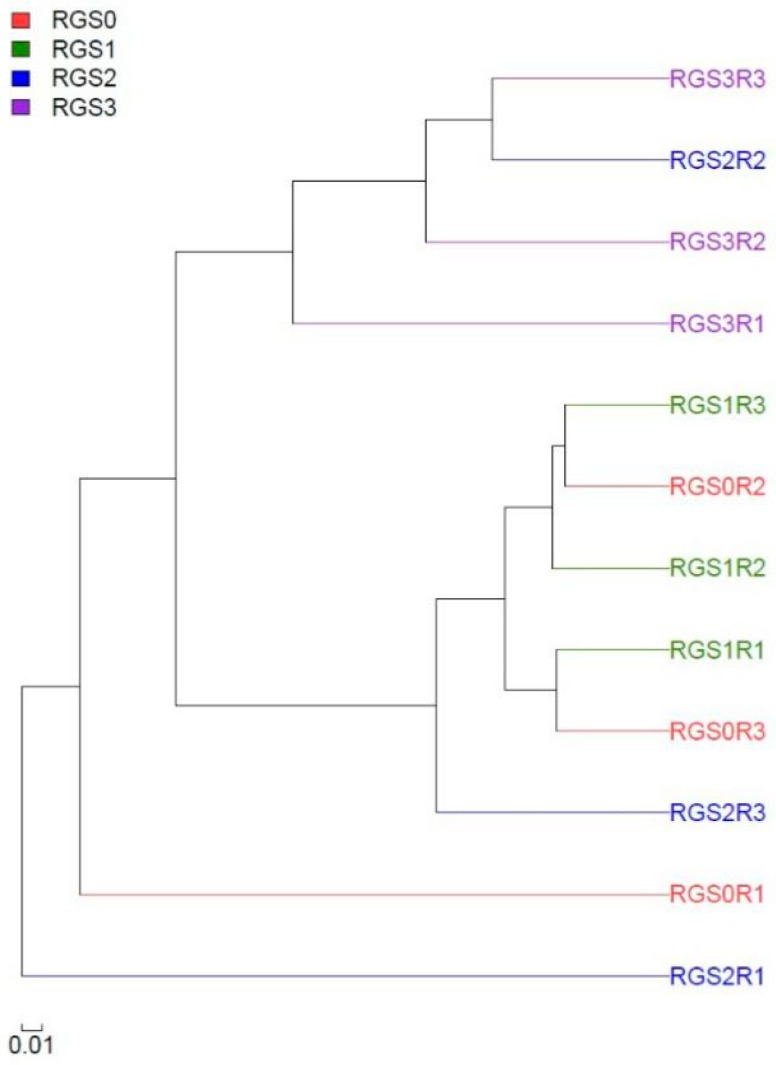
Hierarchical cluster analysis of replications of different RGS treatments on the basis of fungal groups’ presence.

Bray–Curtis similarity distance matrix-based hierarchical cluster analysis (HCA) was performed for all the replications of the three treatments and RGS0 (Figure 7). Two major groups were formed, with different replications of various treatments in each cluster. Moreover, replication 1 of both RGS0 and RGS1 were more dissimilar to the rest of replications due to their unique abundance levels of fungal taxa. Generally, the dendrogram showed similar grouping patterns for RGS1 and RGS0, while most of the replications of RGS2 and RGS3 were clustered together.

### 2.10. Correlation between Soil Chemical Characteristics and Soil Microbial Community

**Figure 8:**
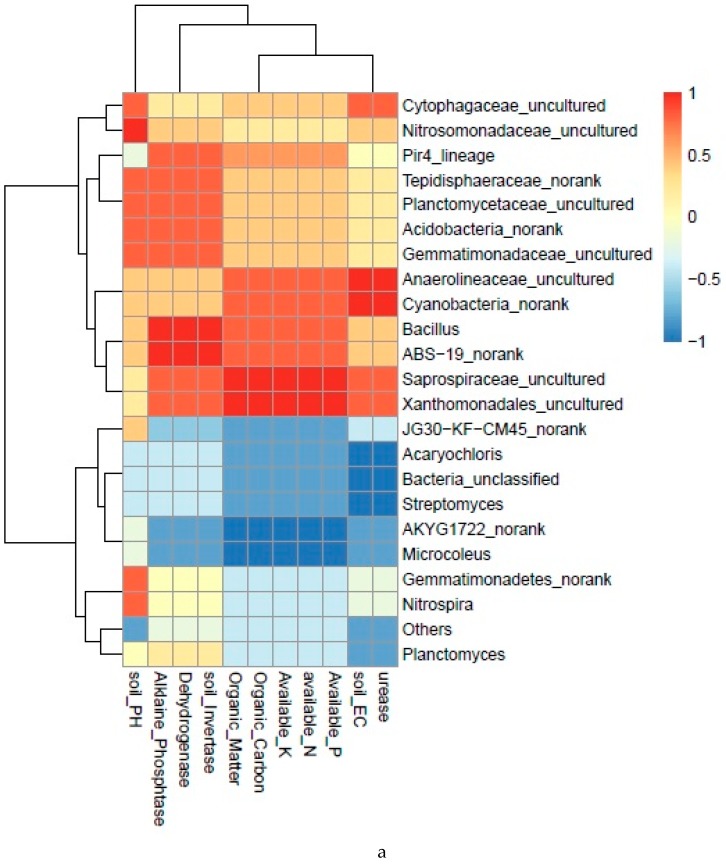

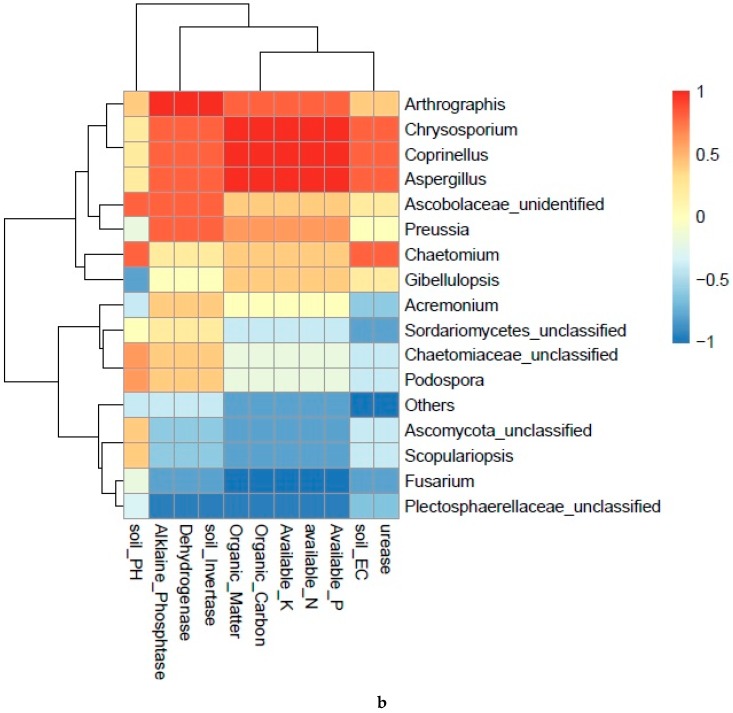
Correlation analysis of soil chemical properties with the presence of bacterial (**a**) and fungal (**b**) communities.

The Spearman’s rank correlation analysis was calculated among all the chemical properties, and taxon abundance levels for bacteria and fungi, and values were illuminated in heat maps (Figure 8a,b). Highly significant correlations were observed between various soil properties, enzyme activities, and microbial community taxa. A highly significant positive correlation was observed between chemical properties, i.e., available N, P, K, organic carbon, organic matter, soil pH, EC, soil dehydrogenase, invertase and alkaline phosphatase, and bacterial taxa, i.e., Anaerolineaceae, Cyanobacteria, *Bacillus*, *ABS*-19, Pir4, Gemmatimonadaceae, Saprospiraceae, and Xanthomonadales (Figure 8a). Soil dehydrogenase and invertase activity had a strong positive association with the abundance levels of *Bacillus*, *ABS*-19, Pir4, Gemmatimonadaceae, Planctomycetaceae, Saprospiraceae, and Tepidisphaeraceae. Likewise, a higher abundance of the uncultured members of Anaerolineaceae, Cyanobacteria, Cytophagaceae, and Xanthomonadales correlated with the increased activity of soil urease and alkaline phosphatase in the raw garlic stalk treatments. Moreover, a significantly negative correlation was observed between soil characteristics, e.g., level of soil nutrients, organic matter, available carbon, and enzyme activity, and bacterial taxa, i.e., JG30-KF-CM45, *Planctomyces*, Gemmatimonadetes, *Nitrospira*, *Streptomyces*, AKYG1722, *Microcoleus*, and *Acaryochloris*.

Certain fungi taxa, including *Coprinellus*, *Aspergillus*, Ascobolaceae, *Chaetomium*, *Arthrographis Chrysosporium*, and *Preussia*, exhibited a strong positive correlation with soil properties such as available N, P, K, carbon, organic matter, soil pH, EC, and the activity of soil dehydrogenase, invertase, urease, and alkaline phosphatase (Figure 8b). The genus *Chaetomium* and the family Ascobolaceae were found to be positively correlated with soil pH, EC, soil dehydrogenase, invertase, and alkaline phosphtase. On the other hand, a highly significant negative correlation was found between soil chemical properties and the abundance levels of Ascomycota, *Fusarium*, *Scopulariopsis*, and Plectosphaerellaceae. By far, *Fusarium* showed the highest negative association with the increased concentration of nutrients, pH, EC, and enzyme activities in RGS-amended soils.

## 3. Discussion

Traditional monocropping deteriorates soil health, leading to the reduction of crop productivity due to a complex interplay of various consequential factors. Continuous cropping affects the soil biological and chemical characteristics, aggravating soil-borne diseases and imparting a negative influence on the microbial population in the root zone [6]. Numerous remedial procedures are in practice for preserving and reclaiming soil health. We previously reported that RGS application affects growth by enhancing the antioxidant defense system and reducing the incidence of *Fusarium* wilt [10]. Therefore, in the present study we investigated the in situ impact of RGS amendment on the soil chemical properties and the soil microbiome in an eggplant production system in monoculture under plastic greenhouses in China, which has not been reported before.

### 3.1. Effect of RGS on Soil Chemical Properties

The application of organic material into soil is a widely adopted strategy to alleviate soil degradation [1,3]. Raw stalks improve soil biological and chemical properties directly as well as indirectly, subsequently leading to the enhancement of the crop yield. Soil pH profoundly influences nutrient availability [3], nutrient adsorption, soil microbial activity [3], and enzyme activity [12]. In the present study, increasing the RGS dose reduced soil pH and increased EC (Table 2). These findings are in agreement with the results of previous studies [4,22,23], which also demonstrated changes in pH due to amendments with different plant residues. Changes in pH and EC due to RGS amendment might be due to the release of acidic compounds from RGS. It has been reported that during decomposition, crop residues release compounds that may increase the amount of ions in soil, resulting in increased soil EC [24]. The application of different organic amendments to soil induces increases in levels of C, N, P, and K [12], and such enhancements in the available nutrients resulting from the mineralization and transformation of RGS were also observed in the present study. Lower soil pH may be associated with higher nutrient availability and subsequent uptake by roots [25]. As observed in our study, RGS2 and RGS3 had the lowest pH with higher concentrations of nutrients. Moreover, improved N, P, and K availability may influence the soil microbes and enzyme activity, thus playing a vital role in the improvement of soil fertility [12].

Soil enzymes are important indicators of soil health and nutrient cycling, eliciting a quick response to management strategies. Increased invertase, dehydrogenase, and urease activity are indicative of higher nitrogen and carbon transformation in soil [3,26]. Similarly, phosphatase has a positive association with carbon, nitrogen, and organic phosphorus contents as well as with soil pH [24]. Our results showed that urease, invertase, dehydrogenase, and phosphatase activities increased with increasing RGS concentration. The application of RGS decreased pH and increased urease activity. Jeong et al. [27] demonstrated that urease activity is associated with soil pH with an ideal range of 7–8.2, which is in agreement with our results. Moreover, increased dehydrogenase and phosphatase activity in RGS-amended soils corroborate the findings of Sial et al. [3,4].

### 3.2. Effect of RGS on Soil Microbial Communities

#### 3.2.1. Bacterial Abundance and Community Structure

Soil biochemical changes may be associated with changes in bacterial community structures, abundance, and species richness. High-throughput sequencing systems were employed to characterize the microbial communities and to ascertain their taxonomies through quality sequence data obtained from relatively less abundant DNA of soil microbes present in the environment [28,29]. In recent years, Illumina MiSeq sequencing techniques have been used by researchers to study soil microbial diversity, community structure, species evenness, and richness around the rhizosphere [30,31]. Wang et al. [32] also studied the relationship between soil microbial structure and organic farming through Illumina MiSeq sequencing approaches. In the present study, a similar approach was used to investigate the effect of RGS treatment on the soil bacterial community diversity, and the abundance of important taxa across different treatments. Higher bacterial diversity in soil may help in overcoming soil-borne pathogens, due to antagonism and competition for the host within the community [33]. The bacterial abundance of the taxa Anaerolineaceae, Acidobacteria, Cyanobacteria, Cytophagaceae, *Bacillus*, *JG30-KF-CM45*, *ABS*, *Planctomyces*, Gemmatimonadetes, *Pir4*, Gemmatimonadaceae, and Nitrosomonadaceae was higher in all the treatments. Similar abundance levels of these species were also observed by Li et al. [6] in eggplant monocrop soil of Heilong Jiang Province, China. The abundance of these taxa may be due to identical soil conditions or the effect monocropping of eggplant. Our results are in line with the previous investigation of Jiang et al. [34], who observed Nitrosomonadaceae, Anaerolineaceae, and Acidobacteria as the dominant bacteria in the apple rhizosphere in the Bohai Gulf of China. Across treatments, bacterial abundance showed peculiar patterns, where RGS0 had a higher abundance of diverse species, as evident from LEfSE analysis. RGS0 had a higher bacterial content of Anaerolineaceae (*Chloroflexi*) and Thiotrichaceae, and a lower abundance of Saprospiraceae (0.68%), Tepidisphaeraceae (0.70%), and Xanthomonadales (0.52%). Similar results depicting a reduction in *Chloroflexi* due to the application of organic material were reported by Trivedi et al. [35]. The application of RGS promoted the growth of some beneficial microorganisms, sharing an antagonistic effect on soil-borne pathogens and indicating improved plant growth due to the release of active metabolites, e.g., rhizobacteria *Bacillus*, which is capable of fighting against phytopathogens by releasing active secondary metabolites [34]. Our results were supported by a previous study [36]. Xanthomonadales are known to be hydrocarbon decomposers, and have the ability to obtain carbon from co-existing microorganisms [37]. Furthermore, other beneficial functions of soil microbes include the decomposition of wood material (Xanthomonadaceae) [38,39], bio-control agents against citrus canker (*Pseudoxanthomonas*) [22], and nitrogen uptake (*Chryseolinea*) [40], resulting in increased growth. Previously, it was reported that these species act as plant growth stimulants and an increase in their abundance was observed due to amendment of *Brassica* residue into the soil of a continuous eggplant monocrop system [6].

Anaerolineaceae fermentatively utilize sugars and proteinaceous compounds, while Thiotrichaceae are known for their sulphur cycling capabilities [41]. The content of Cytophagaceae was higher in RGS1 *Pir4*_lineage, and that of *Acaryochloris* was lower than other treatments. Members of the *Cytophaga* genus are known to be proficient at digesting insoluble cellulose, like members of the genus *Sporocytophaga*, its close relative. Cellulose utilization in some of the *Cytophaga* members seems to involve a novel collection of glycosyl hydrolases [42]. RGS2 had a relatively higher abundance of *ABS*-19 and Tepidisphaeraceae, and a significantly lower presence of Cyanobacteria. The abundance levels for Cyanobacteria, *ABS*-19, Saprospiraceae, and Xanthomonadales in RGS3 increased compared to RGS0. Cyanobacteria include symbiotic nitrogen-fixing bacteria of the genera *Azorhizobium*, *Allorhizobium*, *Bradyrhizobium*, *Rhizobium*, *Mesorhizobium*, and *Sinorhizobium*, which not only help in nodulation, but also have a positive effect on the yield of legume crops [43]. Moreover, *Microcoleus* (*Cyanobacteria*) and *Acaryochloris* abundance was significantly reduced in RGS3 treatment. Likewise, a considerable decrease in bacterial abundance was observed from RGS0 to RGS3 for Anaerolineaceae (87%), *AKYG1722* (66%), and *Streptomyces* (56%). *Streptomyces* is known to produce various antifungal compounds and can act as a biocontrol agent for different plant diseases. Thus, a reduction in the relative abundance of *Streptomyces* could be indicative of reduced inhibition of fungal growth [44]. Previously, declines in certain bacterial species as a result of changes in soil conditions due to residue treatments or possible antagonism have been reported [6,32,34].

#### 3.2.2. Fungi Abundance and Community Structure

In our study, seven fungal taxa, i.e., *Chaetomium*, *Coprinellus*, *Acremonium*, *Fusarium*, *Aspergillus*, Sordariomycetes, and Plectosphaerellaceae, were abundantly found in the soil, which was consistent with earlier reports [12,45]. However, analyses confirmed that soil chemical properties and RGS treatments were decisive factors altering the fungal community in the soil. This was exhibited by sharp decrease in some taxa, i.e., Ascomycota, *Fusarium*, *Acremonium*, and Sordariomycetes. Ascomycota, a dominant fungus phylum, includes many plant pathogens and is considered a vital source of numerous toxins [46,47]. *Chaetomium* is an important genus of the class Sordariomycetes, which acts as a cellulolytic organism and easily degrades paper and fabrics [48]. In our experiment, *Chaetomium* was reduced with increasing RGS concentration. This might be attributed to its sensitive nature to phenolics [49]. During decomposition, RGS releases allelochemicals that might be responsible for reducing *Chaetomium* in raw-garlic-stalk-amended soil. Pandey [50], while studying fungal diseases, reported *Fusarium solanias* to be one of the major pathogenic fungi causing eggplant diseases. The reduction of *Fusarium* due to treatments of raw garlic stalk showed the utilization of garlic residue to be beneficial for preventing *Fusarium oxysporum*, and hence the *Fusarium* wilt disease in eggplant. Similarly, in the present study, endophytic plant pathogens belonging to the genus *Acremonium* and the class Sordariomycetes were significantly reduced by RGS addition [46,51].

In addition, *Acremonium* species are known to cause crown rot disease in different fruits and vegetables [52]. On the contrary, an increase in the abundance of some beneficial fungal genera such as *Coprinellus*, *Aspergillus*, *Gibellulopsis*, and *Chrysosporium* was observed in RGS2 and RGS3. Meanwhile, *Preussia* and *Podospora* were increased in RGS2. *Preussia* spp. promote plant growth due to their ability to release indole acetic acid (IAA) [53]. *Podospora* spp. suppress *Fusarium* disease in soil [54]. *Coprinellus* includes a diverse group of fungi capable of suppressing soil-borne pathogens, e.g., a strain of *Coprinellus curtus* has been found to be effective against bottom-rot disease in Chinese cabbage. In addition, antagonism with *Fusarium* through hyphal interaction suppresses root-rot disease and *Fusarium* wilt in tomato and melon, respectively [55]. Some species of *Aspergillus*, such as *Aspergillus niger*, have been reported to exhibit strong antibacterial activity against several plant pathogens such as *Fusarium oxysporum f.* sp. [56]. Kawaradani et al. [57] studied the effect of *Gibellulopsis chrysanthemi* on six vegetables, including eggplant, and reported that the fungus causes diseases in asteraceous plants, whereas vegetables remained resistant against its virulence. However, some species, e.g., *Verticillium nigrescens* of the genus *Gibellulopsis*, could be pathogenic to eggplant [58]. *Chrysosporium pseudomerdarium* produces gibberellins, and thus might promote plant growth [59]. A higher abundance of these pathogens can be attributed to the altered chemical properties of the soil as well as the decomposition of the residue [51]. The higher eggplant yield in RGS2 can be associated with the favorable chemical properties of the soil, the low occurrence of *Fusarium* wilt, and the higher diversity of the plant fungal population as a result of RGS incorporation. This agrees with the previous findings of Van et al. [60], suggesting that microbial diversity acts as a crucial factor for reducing the pathogen populace in soil.

## 4. Materials and Methods

### 4.1. Experiemtnal Description and Soil Sampling

Pot experiments were performed and eggplants were grown from March 2016 to October 2017 under a plastic tunnel located at the research station of Northwest A&F University, Yangling, Shaanxi, China, as reported by our previous report by Ghani et al. [10]. Briefly, experiments were conducted with 4 treatments: (i) Control (RGS0); (ii) incorporation of 1% RGS (RGS1); (iii) 3% RGS (RGS2); and (iv) 5% RGS (RGS3) into 100g of soil. The experiments were performed in a completely randomized design with three replications. The RGS was collected and prepared as reported previously [10]. The basic characteristics of soil and raw garlic stalk were investigated [10] and are summarized in Table S2.

After harvesting eggplants, fresh soil samples were collected from each pot in 2016 and 2017. The pot soil sampling criteria for microbial analysis were those previously followed in Reference [61]. In brief, soil samples were immediately stored in an icebox and taken to the laboratory. The soil was thoroughly homogenized (2 mm sieved); and divided into two sub-samples. One sub-sample was air-dried for soil chemical properties, another sub-sample was saved in sterilized falcon tubes and stored at −80 °C for DNA extraction. Soil chemical properties were analyzed for the sub-samples from both experiments conducted in 2016 and 2017, while molecular analyses (Illumina MiSeq sequencing) were done for the sub-samples from the pot assays conducted in 2017.

### 4.2. Soil Chemical Analyses

Soil pH was determined by pH meter using 1:1 water:soil suspension (PHS-3C, LIDA, Shanghai, China). EC (µs cm^−1^), was measured by microprocessor conductivity meter (DDS-12DW, Xiaoshan, China by taking soil and distilled water (*w*/*v*) in a 1:5 ratio. Total nitrogen, organic carbon, and C:N of RGS were measured with a CN Analyzer (Vario Max, Elementar, Germany). Soil organic carbon was measured by the oxidation method with 1N K_2_Cr_2_O_7_ and 98% H_2_SO_4_ solution, followed by titration with 0.2 N FeSO_4_ [62]. Total nutrients (N, P, K) of RGS were determined colorimetrically by digesting samples in H_2_SO_4_ and HClO_4_, as described by Reference [63]. Available phosphorous in soil was determined following Olsen et al. [64] in a wet digestion through the calorimetric method of molybdenum antimony resistance, and absorbance was taken at 880 nm using a spectrophotometer (UV-VIS spectrophotometer, Model UV-2450, Shimadzu, Kiyoto, Japan). Available K was extracted using a neutral solution of NH_4_OAc (1 mol L^−1^) followed by emission spectroscopy (FP 6410, Shanghai Bante Instrument Co., Ltd., China) [65].

### 4.3. Assessment of Soil Enzyme Activities

The activities of soil invertase, urease, and alkaline phosphatase were determined by quantifying the release of glucose, NH_3_–N, and P_2_O_5_ products in soil samples. Soil solutions of 8% sucrose, 10% urea, and 0.5% disodium phenyl phosphate were used as the substrate, followed by incubation at 37 °C for 24 h. Absorbance was recorded using spectrophotometry (UV-VIS spectrophotometer, Model UV-2450, Shimadzu, Kiyoto, Japan) at 508, 578, and 660 nm [66]. Dehydrogenase activity was evaluated using a colorimetric method based on the determination of triphenyl formazan (TPF) produced after the reduction of 2,3,5-triphenyltetrazolium chloride (TTC) [67] spectrophotometrically (UV-VIS spectrophotometer, Model UV-2450, Shimadzu, Kiyoto, Japan) at 485 nm.

### 4.4. Microbial DNA Extraction and PCR Amplification

DNA was extracted from 0.5 g of soil using an E.Z.N.A. ^®^ Soil DNA Extraction Kit (Omega Bio-tek, Norcross, GA, USA) as instructed by the manufacturer protocol. The purity and quantity of the DNA were assessed using a NanoDrop 2000 UV–vis spectrophotometer (Thermo Scientific, Wilmington, DE, USA) and agarose gel electrophoresis. The bacterial V3–V4 regions of 16S rRNA were amplified using 515F (GTGCCAGCMGCCGCGG) and 907R (CCGTCAATTCMTTTRAGTTT) primers. Similarly, the fungal internal transcribed spacer (ITS) region of 18S rRNA was amplified using ITS1F (CTTGGTCATTTAGAGGAAGTAA) and ITS2R (GCTGCGTTCTTCATCGATGC) primers.

Triplicate PCR reactions were performed in a 20 μL master mix containing 10 ng of template DNA, 4 μL of 5 × Fast Pfu buffer, 0.4 μL of Fast Pfu Polymerase, 2 μL of 2.5 mM dNTPs, and 0.8 μL of primer (5 μM). The PCR reaction profile was performed with denaturation at 95 °C for 3 min, 27 cycles of 30 s at 95 °C, 30 s of annealing at 55 °C, 45 s of elongation at 72 °C. and a terminal extension at 72 °C for 10 min. The amplicons were resolved on a 2% agarose gel, and gel extraction was performed using a AxyPrep DNA Gel Extraction Kit (Axygen Biosciences, Union City, CA, USA). The extracted DNA was quantified using QuantiFluor™ ST (Promega, Madison, WI, USA).

### 4.5. Profiling of Illumina MiSeq Analyses

After purification, the amplicons were pooled in equimolar ratios and sequenced (2 × 300 bp) according to standardized protocol by Majorbio Bio-Pharm Technology Co. Ltd. (Shanghai, China) using an Illumina MiSeq platform (Illumina, San Diego, CA, USA). Post sequencing demultiplexing of raw paired reads was performed. The ambiguous and unidentified sequences were removed using the UCHIME method (version 4.2.40, San Francisco, CA, USA). Raw reads were filtered against quality by Trimmomatic and merged by FLASH. High-quality sequences were assigned into operational taxonomic units (OTUs) at 3% phylogenetic distance using the UPARSE method (version 7.1, http://drive5.com/uparse/, accessed date: 25 April 2019). The RDP (Ribosomal Database Project) Classifier algorithm (http://rdp.cme.msu.edu/, accessed date: 25 April 2019) was used to analyze the taxonomy of each 16S rRNA gene and ITS fungal sequence against the Silva (SSU123) database at a 70% threshold confidence level. Bacterial and fungal community diversity, community composition, OTU richness, alpha diversity indices, and overlapping sequences were calculated in the vegan package in R (Version 2.15.2, vegan package, Foundation for Statistical Computing, Vienna, Austria). The Spearman’s rank correlation analysis was performed as a heat map figure to assess the relationship between the soil microbial class and environmental factors.

### 4.6. Statistical Analysis

Two-way analysis of variance (ANOVA) with two factors factorial arrangement (treatment × year) was executed as statistical analysis using SPSS software (version 16.0., SPSS, Inc., Chicago, IL, USA) to estimate the soil chemical characteristics. Mean separation among treatments was examined using the least significant difference (LSD) test at *p* < 0.05. The Spearman’s rank correlation analysis was illuminated as a heat map figure to assess the relationship between soil microbial class and environmental factors. PCA and HCA were performed to reveal the differences among different soil samples. The significant changes in OTUs were visualized using rarefaction curves and Venn diagram analysis. The linear discriminant analysis (LDA) effect size (LEfSE) algorithm (https://huttenhower.sph.harvard.edu, accessed date: 25 April 2019) was performed to show the significant identification differences in microbial communities based on different taxonomic ranks. An α-value for the factorial Kruskal–Wallis test was set at 0.05, and the threshold for the logarithmic LDA score was 2.0.

## 5. Conclusions

This study revealed soil microbial community dynamics in response to the application of RGS. These results also support the idea that the previously shown dose-dependent impact of RGS preventing the soil-quality-related constraints in the monocultured eggplant under plastic greenhouses might be related to the changes in the soil biochemical and microbial communities upon the treatment of RGS. In brief, a higher amount of RGS application significantly induced soil nutrient cycling and biological changes by lowering the pH level in consecutive years. Such soil modification can directly shape the soil microbiome and diversity structures. However, the microbial communities characterized in this study were complex and dynamic with respect to the exogenous organic input, plant response, and soil condition, as well as the plant species. In particular, the RGS amendments effectively influenced the soil microbial communities of both the beneficial and harmful species, affecting their richness and abundance, as compared to the control. Therefore, from the present study it may be advocated that the application of appropriate RGS to eggplant mono-cropping can be exploited to avoid the deleterious effects induced by pathogenic fungi. However, further studies are required to evaluate the molecular and genetic modulations in the eggplant by RGS amendment.

## Figures and Tables

**Table 1:** Influence of raw garlic stalk on soil pH, electrical conductivity (EC), organic carbon, and organic matter (mean ± standard error; *n* = 3).

Treatment	pH			EC (µs⋅cm^−1^)			Organic Carbon (g⋅kg^−1^)			Organic Matter (g⋅kg^−1^)		
	2016	2017	Means	2016	2017	Means	2016	2017	Means	2016	2017	Means
RGS0	7.89	7.91	7.90a	164.24	162.52	163.41b	15.21	16.09	15.66c	26.22	27.76	26.99c
RGS1	7.85	7.80	7.82b	228.50	230.67	229.58a	19.27	20.36	19.18b	33.21	35.10	34.16b
RGS2	7.78	7.76	7.77bc	227.00	235.83	231.42a	21.46	23.02	22.24a	37.00	39.70	38.35a
RGS3	7.75	7.70	7.72c	234.00	238.67	236.33a	23.06	24.17	23.62a	39.76	41.68	40.72a
Year means	7.82	7.79		213.43	216.94		19.75	20.916		34.04	36.06	
LSD-test	Treatment	Year	Interaction	Treatment	Year	Interaction	Treatment	Year	Interaction	Treatment	Year	Interaction
	***	NS	NS	***	NS	NS	***	NS	NS	***	NS	NS

Different letters indicate significant differences between means within columns at *p* < 0.05 by least significant difference (LSD) test. *** *p* < 0.001 by one-way analysis of variance (ANOVA). NS: non-significant.

**Table 2:** Influence of raw garlic stalk on soil available nutrients (mean ± standard error; *n* = 3).

Treatment	Available N (mg⋅kg^−1^)			Available P (mg⋅kg^−1^)			Available K (mg⋅kg^−1^)		
	2016	2017	Means	2016	2017	Means	2016	2017	Means
RGS0	70.79	72.20	71.50b	194.44	203.11	198.78c	226.33	236.67	231.50b
RGS1	75.13	78.13	76.63b	198.55	204.78	201.66c	232.17	248.33	240.25b
RGS2	119.10	125.00	122.05a	214.55	227.85	221.20b	377.83	385.50	381.67a
RGS3	120.24	126.94	123.59a	221.85	232.15	227.00a	396.83	403.83	400.33a
Year Means	96.31a	100.57a		207.35	216.97		308.29a	318.58a	
LSD-test	Treatment	Year	Interaction	Treatment	Year	Interaction	Treatment	Year	Interaction
	***	NS	NS	***	NS	NS	***	NS	NS

Different letters indicate significant differences between means within columns at *p* < 0.05 by LSD test. *** *p* < 0.001 by ANOVA. NS: non-significant.

**Table 3:** Influence of raw garlic stalk on soil enzymes activities (mean ± standard error; *n* = 3).

Treatment	Soil Invertase Activity (Glucose mg g^−1^)			Soil Urease Activity (NH_3_-N mg.g^−1^)			Soil Dehydrogenase (mg TPF kg^−1^ soil h^−1^)			Soil Phosphatase (P_2_O_5_ mg.100g^−1^)		
	2016	2017	Means	2016	2017	Means	2016	2017	Means	2016	2017	Means
RGS0	88.77	91.75	90.26d	1.67f	3.19c	2.43	15.61	16.21	15.91c	5.80e	6.12de	5.96d
RGS1	101.85	110.30	106.07c	2.10e	4.69b	2.40	19.26	19.74	19.50b	6.77cd	7.23c	7.00c
RGS2	116.61	118.00	117.30b	2.79d	4.88ab	3.83	29.20	31.52	30.27a	8.52b	9.26a	8.89a
RGS3	129.31	134.88	132.10a	3.03c	5.08a	4.06	24.59	25.78	25.18b	8.01b	8.66ab	8.34b
Year means	109.13	113.73		2.40	4.46		22.163	23.27		7.28	7.81	
LSD-test	Treatment	Year	Interaction	Treatment	Year	Interaction	Treatment	Year	Interaction	Treatment	Year	Interaction
	***	NS	NS	***	***	***	***	NS	NS	***	*	NS

Different letters indicate significant differences between means within columns at *p* < 0.05 by LSD test. * *p* <0.05, *** *p* < 0.001 by ANOVA. NS: non-significant.

## Supplementary Materials

The following are available online at https://www.mdpi.com/1422-0067/20/9/2125/s1.
