# Supplementary material for: Changes in the Soil Microbiome in Eggplant Monoculture Revealed by High-Throughput Illumina MiSeq Sequencing as Influenced by Raw Garlic Stalk Amendment
Source: Int J Mol Sci. 2019 Apr 29;20(9):2125. doi: 10.3390/ijms20092125 (PMC6539610; doi:10.3390/ijms20092125)
Supplement: Supplementary file 1 [file ijms-20-02125-s001.pdf]

Table S1. OTUs' Diversity Indices for bacterial and fungal reads (S.E n = 3).

	Treatment	OTUs Number	Ace	Chao richness estimator	Shannon diversity index	Simpson
Bacteria	RGS0	2451±20	3047±36	3027±19	6.43±0.02	0.006±0.0006
	RGS1	2450±14	3010±39	2987±45	6.38±0.12	0.01±0.0002
	RGS2	2484±32	2945±10	2964±91	6.61±0.02	0.003±0.0002
	RGS3	2419±42	2976±61	3008±87	6.29±0.16	0.014±0.008
Fungi	RGS0	186±23	242±16	228±20	1.63±0.22	0.452±0.12
	RGS1	179±12	219±2	212±5	1.37±0.14	0.569±0.03
	RGS2	198±12	251±4	241±8	2.05±0.48	0.298±0.11
	RGS3	182±16	221±18	218±18	1.85±0.08	0.292±0.16

Table 2. Characteristics of basic soil and RGS used in the experiment (S.E n = 3).

Original soil properties		Garlic stalk properties
Soil Type	Brown -loamy-alkaline-Orthic Anthrosol	
pH	7.75 ± 0.5	7.45 ± 0.6
EC (μS cm^{-1})	383 ± 6	620 ± 10.5
Organic Carbon (g.kg^{-1})	15.13 ± 0.9	370.08 ± 8.5
Total Nitrogen (g.kg^{-1})	1.14 ± 0.06	2.07 ± 0.5
C:N	13.42 ± 0.8	178.78 ± 7.5
Organic Matter (g.kg^{-1})	12.97 ± 0.5	639.25 ± 12,5
Total Phosphorus (g.kg^{-1})	0.91 ± 0.03	12.87 ± 1.5
Total Potassium (g.kg-1)	6.87 ± 0.5	0.19 ± 0.05
Available N (mg.kg^{-1})	60.3 ± 3.5	-
Available P (mg.kg^{-1})	55.01 ± 5.1	-
Available K (mg.kg^{-1})	189 ± 4.8	-
Soil Planting History	Eggplant continuously planted for four years	
